# lncScore: alignment-free identification of long noncoding RNA from assembled novel transcripts

**DOI:** 10.1038/srep34838

**Published:** 2016-10-06

**Authors:** Jian Zhao, Xiaofeng Song, Kai Wang

**Affiliations:** 1 Department of Biomedical Engineering, Nanjing University of Aeronautics and Astronautics, Nanjing 210016, China; 2 Zilkha Neurogenetic Institute, Keck School of Medicine, University of Southern California, Los Angeles, CA 90089, USA; 3 Division of Bioinformatics, Department of Preventive Medicine, Keck School of Medicine, University of Southern California, Los Angeles, CA 90089, USA; 4 Institute for Genomic Medicine, Columbia University Medical Center, New York, NY 10032, USA; 5 Department of Biomedical Informatics, Columbia University Medical Center, New York, NY 10032, USA

## Abstract

RNA-Seq based transcriptome assembly has been widely used to identify novel lncRNAs. However, the best-performing transcript reconstruction methods merely identified 21% of full-length protein-coding transcripts from H. sapiens. Those partial-length protein-coding transcripts are more likely to be classified as lncRNAs due to their incomplete CDS, leading to higher false positive rate for lncRNA identification. Furthermore, potential sequencing or assembly error that gain or abolish stop codons also complicates ORF-based prediction of lncRNAs. Therefore, it remains a challenge to identify lncRNAs from the assembled transcripts, particularly the partial-length ones. Here, we present a novel alignment-free tool, lncScore, which uses a logistic regression model with 11 carefully selected features. Compared to other state-of-the-art alignment-free tools (e.g. CPAT, CNCI, and PLEK), lncScore outperforms them on accurately distinguishing lncRNAs from mRNAs, especially partial-length mRNAs in the human and mouse datasets. In addition, lncScore also performed well on transcripts from five other species (Zebrafish, Fly, C. elegans, Rat, and Sheep). To speed up the prediction, multithreading is implemented within lncScore, and it only took 2 minute to classify 64,756 transcripts and 54 seconds to train a new model with 21,000 transcripts with 12 threads, which is much faster than other tools. lncScore is available at https://github.com/WGLab/lncScore.

Over the past decades, a large number of studies have revealed that non-coding RNAs are pervasively expressed in eukaryotic genome, and that they are not junk RNAs but functional RNA molecules^1^,^2^,^3^,^4^,^5^,^6^,^7^. Beyond the short ncRNAs (e.g. miRNA, siRNA, and piRNA), there are growing interests to study the poorly understood, yet the most common long noncoding RNA (lncRNA) species with a length larger than 200 nt^8^,^9^. Although only a small fraction of them have been functionally validated, lncRNAs seem to play important roles in various critical biological process, such as chromatin remodeling, genomic splicing, transcription, translation, epigenetic regulation, cell proliferation and differentiation^10^,^11^,^12^,^13^,^14^.

With the development and application of next-generation sequencing techniques, particularly RNA sequencing (RNA-Seq), an increasing number of lncRNAs have been discovered in eukaryotic organisms ranging from nematodes to humans, but there are still a large number of lncRNAs waiting to be found^8^,^15^,^16^,^17^,^18^. For example, in the current GENCODE database, the ratio between the number of protein-coding transcripts and long noncoding transcripts is nearly 3:1 for human genome and about 4:1 for mouse genome^19^. In addition, the scarcity of lncRNAs is quite obvious for plant species, most of which have no lncRNA information as shown in PNRD, the latest plant non-coding RNA database^20^. RNA-Seq has now become a very popular means to detect novel transcripts with the transcriptome assembly software like StringTie^21^, and thousands of novel lncRNAs have been identified from RNA-seq data^22^,^23^,^24^,^25^,^26^. To detect lncRNAs from novel transcripts, the ability for distinguishing coding and long non-coding transcripts becomes very important.

Generally, a simple and rough way to define a candidate transcript to be a lncRNA or not is to check if it contains a long open reading frame (ORF), and if not, then it is most likely a lncRNA. NCBI ORF Finder, ORF Predictor, ESTScan, and framefinder have been used commonly to identify the ORF^27^,^28^,^29^,^30^. The candidates can also be determined as coding or non-coding based on the similarity to known proteins or protein domains (e.g. CPC and PORTRAIT methods^31^,^32^), since ORF-dependent approach usually has a lower accuracy on the prediction of lncRNAs. In terms of sequence conservation across diverse species, lncRNAs exhibit poor conservation compared to mRNAs, which could be applied to identifying lncRNA^33^. PhyloCSF was developed for assessing the coding potential of candidates based on this conservation across the species^34^, whereas iSeeRNA used conservation, ORF and nucleotide sequences-based features with SVM to identify long intergenic non-coding RNA^35^. Besides sequence conservation, mRNAs also tend to show better conservation against characterized proteins, therefore lncRNA-ID calculates the coding potential using a random forest model based on conservation against characterized protein families, ORF and translation scores based on ribosomal coverage^36^. However, the above approaches are based on sequence alignment, either pairwise homology search for similar proteins or multiple alignments to calculate the conservation score, which are extremely time-consuming when processing massive-scale RNA sequencing data. They are also highly dependent on the species that can be compared and the evolutionary history, which may be lineage specific^37^,^38^. Finally, alignment-based methods do not apply to those lncRNAs overlapping with either the sense or antisense strand of coding genes, which cannot be correctly classified by homology searching.

Considering drawbacks of alignment-based methods, some alignment-free methods have been proposed in recent years (Supplementary Table S1). CPAT determined the coding probability of candidates using a logistic regression model built with ORF size, ORF coverage, Fickett TESTCODE statistic, and hexamer usage bias^39^. CNCI extracted five features (i.e. the length and S-score of MLCDS, length-percentage, score-distance and codon-bias) by profiling adjoining nucleotide triplets and also used SVM to distinguish coding and noncoding RNAs^40^. PLEK distinguished lncRNAs from mRNAs through a computational pipeline based on an improved k-mer scheme and SVM algorithm^41^. LncRNA-MFDL identified lncRNAs by fusing multiple features (i.e. open reading frame, k-mer, the secondary structure and the most-like coding domain sequence) and using deep learning classification algorithms^42^. These tools were all tested on full-length testing datasets and proved to have a good classification performance. However, it should be noted that transcripts assembled from RNA-seq data are not all full-length, because it remains a challenge to reconstruct the full-length transcripts from the short reads.

In the previous study about the assessment of transcript reconstruction methods for RNA-seq, it was found that the best-performing methods identified merely 21% of full-length protein-coding transcripts from *H. sapiens* and the detection rate was even lower for noncoding RNAs^43^. It was further found that missing exons severely compromised transcript identification, and greater than 60% transcripts in *H. sapiens* were not identified all of their exons. And even for those transcripts of which all exons had been identified, less than 40% of them were correctly assembled to a full-length annotated splice variant. So those novel transcripts assembled from RNA-seq data are mostly not full-length. Therefore when detecting novel lncRNAs from them, those partial-length protein-coding transcripts, in which start or stop codon was not found (or both of them were not found), are usually misclassified as lncRNAs due to its incomplete CDS, which would decrease the prediction precision of lncRNAs.

In this study, we developed a new powerful alignment-free tool named lncScore using a logistic regression model. To more effectively distinguish long noncoding transcripts from protein-coding transcripts, especially partial-length ones, several new features related to exon and the maximum coding subsequence (MCSS) were used together with ORF-related features in this tool (totally 11 features). Compared with the existing alignment-free tools, lncScore not only performed much better on the human and mouse partial-length datasets, but also had a better performance on the full-length datasets. In addition, lncScore also outperformed them on data from five other species, using the human or mouse classification model. For speeding up data processing, a more efficient multithreading technique was applied in lncScore, resulting in much improved performance over CNCI and PLEK. Thus, lncScore is a fast, accurate, stable and robust tool for detecting long noncoding RNA.

## Materials and Methods

### Data description

High-confidence protein-coding and long noncoding transcripts with the length of >200 nt were downloaded from the human (v.23) and mouse (M6) GENCODE database to build our gold-standard datasets. According to the integrity level of coding sequence (CDS), all of the protein-coding transcripts are classified into two categories: full-length and partial-length protein-coding transcripts. The former are transcripts containing the complete CDS, i.e. initiation codon to termination codon, while ‘partial-length’ is referred to transcripts whose start or stop codon has not been found (or both of them have not been found).

In GENCODE, all the transcripts are classified into three levels according to their annotation types, which also can be regard as three different confidence levels. Transcripts that were manually annotated and verified experimentally by RT-PCR-seq^44^ are highlighted with level 1. Level 2 indicates manually annotated transcripts. As shown in Supplementary Fig. S1, for human datasets, transcripts of level 1 and level 2 were selected respectively to build a training dataset and a full-length testing dataset without partial-length protein-coding transcripts, which were later used to build a partial-length testing dataset with long noncoding transcripts of level 2. For mouse datasets, all of the transcripts of level 1 and part of transcripts of level 2 were used to build the training dataset while remaining transcripts of level 2 were used to build the full-length testing dataset (Supplementary Fig. S2). The partial-length protein-coding transcripts were also removed from the training and full-length testing datasets and then used to build a partial-length testing dataset. To further evaluate prediction performance on other species, transcripts of several other species (Zebrafish, Fruitfly, C. elegans, Rat, and Sheep) were gathered from the Ensembl database (release 82), as shown in Supplementary Table S2^45^. All the transcripts used were longer than 200 nt.

### Logistic Regression

Logistic regression is a supervised learning method and commonly used to model the log odds of dichotomous outcome variables as a linear combination of the predictor variables. Particularly, the predicted values of this model are probabilities and restricted to (0, 1) through the logistic function. Here, the *LogisticRegressionCV* class from a Python module ‘scikit-learn’ was selected to implements logistic regression as it can automatically find out the optimal C in the L2 penalization with built-in cross-validation, and default values were used for all the parameters^46^.

### Sequence features

As an alignment-free method, only those features that can be directly calculated from transcript sequences can be selected. In order to more accurately distinguish lncRNAs from partial-length protein-coding transcripts without prejudice to the classification between lncRNAs and the full-length protein-coding transcripts, totally 11 powerful features were selected in lncScore (Table 1), and they are derived from 3 different groups (e.g. exon, maximum coding subsequences, and ORF).

#### Exon features

Features used in previous alignment-free methods are all derived from the whole transcript sequences, so they would be easily influenced by the incompleteness of transcripts, particularly the partial-length protein-coding transcripts. And, in previous study, it was found that the full-length protein-coding transcripts merely accounts for 21% of all the assembled protein-coding transcripts. Thus, we introduced exon features to the identification of lncRNAs, and only the largest one in each transcript was selected as the representative exon feature. Exon features, as a kind of transcript local features, were less affected by the missing of start or stop codon (or both). In addition, it was found that most of the transcript assembly methods are able to identify more than 70% of coding exons^43^. Therefore, we introduced exon features to the identification of lncRNAs, and only the largest one in each transcript was selected as the representative exon feature.

The location information of exons in each transcript was extracted from the GTF/GFF format files downloaded from GENCODE and ENSEMBL database. GC-content is the proportion of G and C in all bases of a sequence. It has been shown that gene coding regions have a higher GC-content than noncoding sequences^47^. The GC-content was calculated for all the exons in the same transcript, and the maximum one was defined as the exon GC-content. In-frame hexamer frequencies was firstly used to locate coding regions by Claverie *et al*. in 1997^48^, and it was proved to be a powerful feature to discriminating coding transcripts from noncoding transcripts due to the dependence between adjacent amino acids in the proteins^39^. Here, besides the hexamer score, we also defined a hexamer score distance as an extend feature to further distinguish coding regions from noncoding regions. Hexamer score distance was defined as follows:


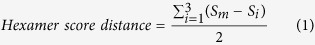


where S_i_ is the hexamer score of the i_th_ reading frame, and S_m_ is the maximum one of them. A nucleotide sequence was scanned three times to generate three forward reading frames, thus the range of *i* is from 1 to 3. Hexamer score and distance were calculated for each exon in a transcript, and then the maximum hexamer score and distance were defined as the exon hexamer score and distance, respectively.

#### Maximum Coding Subsequence

For partial-length protein-coding transcripts, the missing of start or stop codon would directly influence the CDS prediction based on ORF, because ORF is defined as the longest open reading frame and highly related to the start and stop codons. Therefore, the incomplete CDS should be predicted by a method independent of the tart/stop codon. Here, we defined the maximum coding subsequence (MCSS) for partial-length protein-coding transcripts. The MCSS is identified for each transcript sequences by three steps: (1) scanning each transcript to generate three reading frames by beginning with different nucleotides in the first triplet; (2) predicting of the MCSS in each of the three reading frames by applying a modified method based on Kadane’s Algorithm^49^; (3) comparing the coding score of each candidate MCSS from the three reading frames, and defining the maximum one as the best MCSS of the transcript. The procedure of the modified method in step 2 is shown as follows:


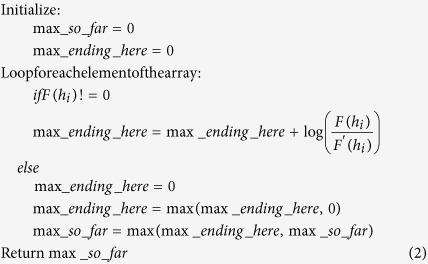


where *h*_*i*_ is the *i*_*th*_ hexamer in the reading frame, and *F(h*_*i*_) and *F’(h*_*i*_) is the frequency of *h*_*i*_ in CDS and noncoding sequences, respectively. For those hexamers not included in any CDS regions, their *F(h)* are zeros and they are commonly beginning with a stop codon (e.g. TAG, TAA, TGA), whereas all kinds of hexamers can be found in noncoding sequences, so their *F’(h)* are all greater than zero.

Besides the coding score, the length and the coding score percentage were also calculated for the best MCSS of the candidate transcript. The coding score percentage was defined as follows:


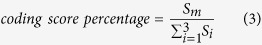


where *S*_*i*_ is the coding score of the MCSS from the *i*_*th*_ reading frame, and *S*_*m*_ is the maximum one of them.

#### ORF

Although the accuracy of the ORF prediction for the partial-length protein-coding transcripts is much lower than that for the full-length ones, several ORF features were also selected to ensure a good performance of lncScore on the classification between lncRNAs and full-length protein-coding transcripts. Here, we extracted six ORF-related features: ORF length, ORF coverage, ORF fickett score, ORF hexamer score and distance. For each transcript, the putative ORF is defined as the longest one of all possible open reading frame in its three forward frames. ORF length is the length of the putative ORF and has been widely used as a fundamental feature to distinguish lncRNA from mRNA. ORF coverage is defined as the ratio of ORF length to transcript length and is commonly used as a complementary feature to the ORF length. Fickett score was at first used to identify protein-coding regions^50^, and then was proved to have a good performance for the classification of protein-coding and noncoding transcripts^39^. ORF hexamer score and distance is similar to exon hexamer score and distance.

### Performance Assessment

Overall accuracy (ACC), sensitivity (S_n_), specificity (S_p_), positive predictive value (PPV), negative predictive value (NPV) and Matthew’s correlation coefficient (MCC) were used to measure the performance of the lncScore^51^,^52^. Receiver operating characteristic (ROC) curve was used to visualize the performance of the binary classifier, and area under the curve (AUC) is used to summarize its performance as a single number. All the performance values were calculated using R with the ROCR package^53^.

### CPAT, CNCI and PLEK setting

lncScore was benchmarked against three other classification programs: CPAT (*version* 1.2.2), CNCI (*version* 2) and PLEK (*version* 1.2), which were installed locally and executed with default parameters. It is worth noting that the ORF coverage is replaced by the transcript length in the CPAT version 1.2.2. In addition, the coding score provided by CNCI is not the probability obtained from the classification model, but a value derived from the S-score of the best MLCDS. Hence, the AUC is not calculated for the CNCI method.

## Results

### Performance on the partial-length testing datasets

**Figure 1:**
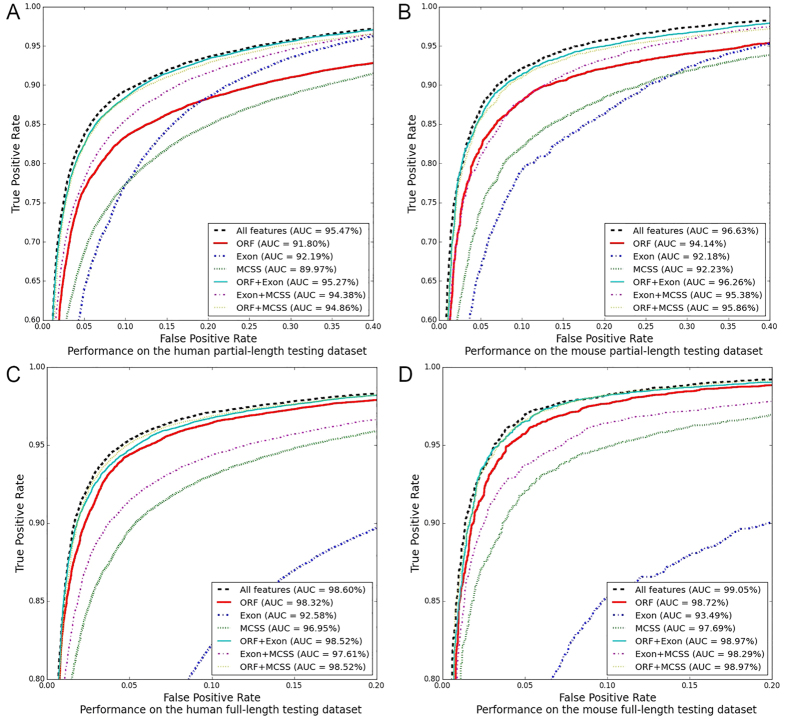
ROC curves of different feature groups on the full- and partial-length testing datasets.

The features used in lncScore can be classified into three groups: exon, ORF, and MCSS (maximum coding subsequence). To evaluate the discriminative power of each feature group, different classification models were created using a single group or multiple groups of features respectively with human and mouse training dataset. The classification performance of each model was then evaluated using ROC curve for the partial-length testing dataset of human and mouse. Figure 1A,B show the performance of lncScore using three groups of features independently or in combination. Each single group of features is able to make a distinction between lncRNAs and partial-length mRNAs. Exon and ORF feature groups are the most distinguishing feature group for human and mouse partial-length testing datasets, respectively. Compared with their performance on the full-length testing datasets, there is a notable decrease of the AUC for the ORF and MCSS feature groups.

The performance of features in each group was evaluated separately using AUC on the partial-length testing dataset and was shown in the Table 2, in which a significant decline can be observed for the performance of lncScore with most features when compared with its performance on the full-length testing dataset. For example, the AUC of ORF length, the best distinguishing feature for the full-length testing dataset, dropped from 97.06%, 97.51% to 83.41%, 83.70% for human and mouse partial-length testing datasets, respectively. In particular, ORF coverage shows a stronger classification ability for partial-length testing datasets than full-length testing datasets, which may be due to the missing of 3′ or/and 5′ UTRs in the partial-length protein-coding transcripts. The exon hexamer score shows a better performance than ORF length and MCSS coding score on the partial-length testing datasets, whereas the latter two features outperform the exon hexamer score and are the best two distinguishing features on the full-length testing datasets. Among all the features, ORF coverage has the highest performance on the mouse partial-length testing dataset, while exon hexamer score performs best on the human partial-length testing dataset, which is consistent with the previous results on the performances of feature groups.

As shown in Fig. 1A,B, combined feature groups shows a better performance than any single feature group within it, and the combination of all the three groups of features leads to the best performance. To further confirm the above results, 10 cross-validations were performed on the training dataset with a single or combined feature group and then the ROC curve was used to measure its classification ability. The result shown in the Supplementary Fig. S3 is similar to that in the Fig. 1. Thus, all the features in the three groups are selected to train the logistic regression models for lncScore. During the training, lncScore presented a 10 cross-validation accuracy of 93.92% and 95.84% on training datasets of human and mouse, respectively. When evaluating the trained model with the human partial-length testing datasets, lncScore correctly predicted 94.67% lncRNAs and 84.15% mRNAs with the default cutoff score 0.5654. Similarly, lncScore showed an accuracy of 94.08% on lncRNAs and 88.39% on mRNAs for the mouse partial-length testing dataset with the default cutoff score 0.4567.

### Performance on the full-length testing datasets

Next, we evaluated the classification performance of lncScore on the full-length testing datasets. Figure 1C,D show the ROC curve of the models trained with one or multiple groups of features for the human and mouse full-length testing datasets. Each one of the three feature groups is capable of distinguishing lncRNAs from full-length mRNAs, and the ORF feature group has the best discriminative power both for human and mouse full-length testing datasets. According to the Table 2, the length in the ORF feature group has the strongest distinguishing ability for the full-length testing datasets, and the next best distinguishing feature is the coding score of MCSS, whose AUC is very close to that of ORF length and their difference is only about 0.005.

Combination of any two groups of features results in better performance than using a single feature group, and combination of the three feature groups can leads to the best performance, which is similar to the results on the partial-length testing datasets and further supports our rationale to use all 11 features. When evaluating the trained models with testing datasets, lncScore correctly predicted 94.67% lncRNAs and 95.56% mRNAs for the human full-length testing dataset with the default cutoff score 0.5654. Similarly, lncScore showed an accuracy of 94.08% on lncRNAs and 97.35% on mRNAs for the mouse full-length testing dataset with the default cutoff score 0.4567.

### Performance comparison on the partial-length testing datasets

**Figure 2:**
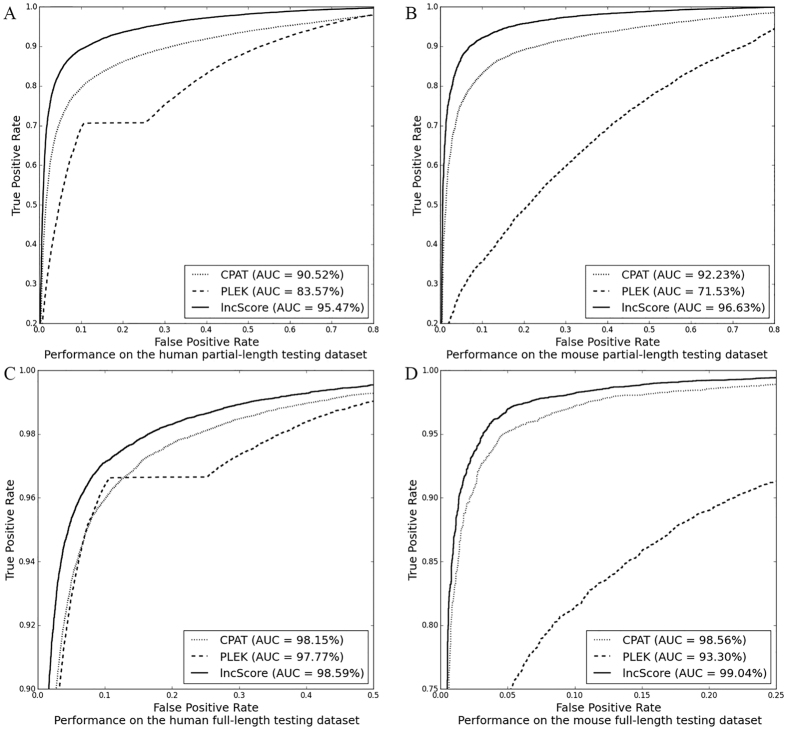
ROC curves of different tools on the full- and partial-length testing datasets.

We compared lncScore with other tools on the human and mouse partial-length testing datasets, and ROC curves of them are shown in Fig. 2A,B. lncScore has the best AUC, which is much higher than that of the other tools for both human and mouse datasets. The plateau observed for the PLEK (Fig. 2A,C) is mainly due to the overlapping lncRNAs between the PLEK training dataset and the human (full- and partial-length) testing datasets, which accounts for 73.69% of lncRNAs in the human testing datasets. Compared with their ROC curves on the full-length testing datasets (Fig. 2C,D), there is obvious performance degradation on the partial-length testing datasets for all the tools, especially the PLEK. The AUC of CPAT, PLEK and lncScore for the partial-length testing datasets of human (mouse) is 7.63% (6.34%), 14.20% (21.78%) and 3.13% (2.42%) lower than that for the full-length testing datasets of human (mouse). The performance degradation is mainly due to the CDS incompleteness of partial-length protein coding transcripts, as the two kind of testing datasets share the same lncRNAs.

Table 3 shows the four assessment indexes (e.g. accuracy, sensitivity, specificity and MCC) of each tested tools on the partial-length testing datasets. It is similar to the results on the full-length testing datasets that lncScore has the best accuracy, sensitivity, NPV and MCC, while PLEK and CNCI has the best specificity respectively for the human and mouse datasets, as shown in Table 3. The specificity of each tools for the partial-length testing dataset is same to that for the full-length testing datasets, because these two testing datasets share the same long noncoding transcripts for human and mouse. However, except the specificity, the other three indexes for the partial-length testing dataset are all lower than those for the full-length testing dataset, which was mainly due to that the incompleteness of partial-length coding transcripts make them more difficult to identify. For the human partial-length testing dataset, the accuracy of lncScore (89.12%) is 5.09%, 8.61% and 25.98% higher than that of CPAT, CNCI and PELK, respectively. For the mouse partial-length testing dataset, the accuracy of lncScore (89.92%) is 10.88%, 13.45% and 39.85% higher than that of CPAT, CNCI and PELK, respectively. These results suggest that lncScore is a reasonably efficient tool for the classification of partial-length coding transcripts and long noncoding transcripts.

### Performance comparison on the full-length testing datasets

Further, we compared lncScore with other three tools (e.g. CPAT, CNCI and PLEK) on the human and mouse full-length testing datasets. As shown in Table 3, lncScore had the best accuracy, sensitivity, NPV and MCC for both human and mouse full-length testing datasets. PLEK had the best PPV and specificity on the human dataset, whereas its other four assessment indexes were lower than those of the other tools. Furthermore, indexes of PLEK were all lower than those of the other tools on the mouse datasets. Compared with CPAT and lncScore, although CNCI had a higher PPV and specificity, its other four indexes were lower. The results above showed the practical classification performance of each tools when using their default cutoffs. To evaluate performances at all cutoff points, AUC was then used to measure the overall performance of each tools.

Figure 2C,D shows the ROC curves of CPAT, PLEK and lncScore, in which lncScore also performs the best. PLEK presented a comparable AUC to CPAT for the human full-length testing dataset, whereas it performed poorly on the mouse full-length testing dataset. Overall, lncScore also showed the best performance on the full-length testing datasets, besides the partial-length testing datasets. These results indicate that lncScore enhanced the ability to distinguish lncRNAs from partial-length protein-coding transcripts without sacrificing the classification performance on the full-length transcripts. Instead, lncScore is slightly more effective than other tools to classify lncRNAs and full-length protein-coding transcripts.

### Performance in cross-species prediction

To evaluate the cross-species predictive power of lncScore, we evaluated lncScore on five other species (e.g. Zebrafish, Fruitfly, C. elegans, Rat, and Sheep) datasets. These species were selected for testing, because their data sets contain well annotated lncRNAs in the Ensemble database. The lncScore models built by human and mouse training datasets were tested individually on these species, and then their performances were measured by comparing with that of three other alignment-free predictors (e.g. CPAT, CNCI, and PLEK). In particular, CPAT has four pre-trained models for human, mouse, fly, and zebrafish, respectively, so we tried to use the specific model (if available) when testing on a specific species. In general, lncScore presents a better performance than other predictors in cross-species predictions (Table 4). More importantly, the mouse model of lncScore even performs better than the CPAT models trained for zebrafish and fly, when tested on zebrafish and fly.

To more objectively assess the performance of lncScore in cross-species prediction, we preformed 10-fold cross validation on datasets for each species with the same features and logistic regression model used in lncScore and then compared their performances with that of lncScore. As shown in Table 4, 10-fold cross validation presents the best classification performance on all the five species, and lncScore performs very closely to the 10-fold cross validation for most of the species, except for sheep, which is a much less well annotated species compared to other model organisms. However, when removing the ORF length from the feature group, new human models of lncScore shows an ACC of 92.19% and an AUC of 96.63% for sheep, while the mean ACC and AUC of 10-fold cross validation on the sheep dataset are 92.71% and 96.96%, respectively. All of these results demonstrate that lncScore can be used to analyze the transcriptome data of other species, and the predictive performance is more influenced by features used in models rather than the training datasets themselves.

### Computational speed

The total computing time of CPAT, CNCI, PLEK and lncScore was measured on the human full-length testing dataset, containing 64,756 transcripts. All these tested tools were run on the same node with two 2.67 GHz Intel X5650 processors, 80 GB memory and Linux operating system. It took CPAT, CNCI, PLEK and lncScore 3.17 m, 2321.86 m, 148.12 m and 21.61 m to process the data with a single thread. However, CNCI, PLEK and lncScore can also be run in a multi-threading manner, and when using 12 threads, the data processing took them 334.54 m, 24.40 m and 2.01 m. These results show that even with 12 threads, CNCI and PLEK are still slower than CPAT and single-threaded lncScore. CPAT is nearly 7-fold faster than single-threaded lncScore, whereas 12-threaded lncScore is approximately 1.5-fold faster than CPAT. Thus, lncScore is especially suitable to process large data sets derived from RNA-Seq.

## Discussion

**Figure 3:**
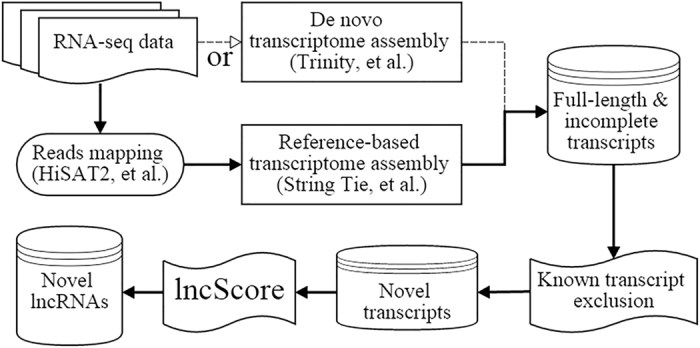
Work flow for identification of novel lncRNAs using RNA-seq data.

The high-throughput RNA-Seq technology has been widely applied to identify novel lncRNAs. As shown in Fig. 3, the general workflow can be divided into three major steps – transcriptome assembly, known transcript exclusion, and lncRNA prediction. It should be noted that incomplete assembled protein-coding transcripts account for 80% or more of the protein-coding transcripts reconstructed by transcriptome assembly tools and the integrity extent of assembled noncoding transcripts is even worse, because it remains a challenge to reconstruct the full-length transcripts using RNA-seq reads^43^,^54^. In the incomplete assembled novel transcripts, incomplete protein-coding transcripts are more incorrectly sorted than incomplete noncoding transcripts. And in the incomplete protein-coding transcripts, the partial-length ones with a fragmentary CDS are more easily misclassified as noncoding transcripts than those containing a complete CDS, which is the key point to distinguish lncRNAs from mRNAs. However, most of the tools available to distinguish lncRNA and mRNA paid less attention to the incomplete transcripts, and only CNCI was tested on a simulated incomplete transcript data set. Protein-coding transcripts used in all of the tools, except for lncRNA-ID, were all selected from RefSeq^55^, which only contains full-length transcripts. Therefore, it is necessary to develop a tool that can efficiently distinguish long noncoding transcripts from the partial-length protein-coding transcripts, not just the full-length protein-coding transcripts.

To establish such a tool, long noncoding transcripts and partial-length protein-coding transcripts were selected from GENCODE, and full-length protein-coding transcripts were also selected. GENCODE is known to have the most complete human and mouse lncRNA annotation to date, and its set of full-length protein-coding transcripts is very similar to RefSeq. In order to distinguish lncRNAs from partial-length mRNAs, several new features were introduced to lncScore, and most of them are unrelated to ORF, such as exon and MCSS features. In all of the features, as shown in the Table 2, exon hexamer score distance shows the most stable performance and exon hexamer score next, whereas exon GC-content shows a significant change. This is largely due to that the hexamer score and distance varies little for exons contained in the CDS of protein-coding transcripts, while GC-content varies more widely. MCSS coding score and its percent show a better performance than most of the other features on either the full-length or partial-length datasets, indicating their robust distinguishing ability. The missing of start/stop codons in the partial-length protein-coding transcript made it harder to accurately predict the ORF. For example, the prediction accuracy of ORF (28.36%) on the human partial-length protein-coding transcripts is much lower than that (94.64%) on full-length ones. So it was supposed that the ORF-related features would have a worse performance than ORF-unrelated features on the partial-length datasets, however, in fact, the former yield a comparable performance with the latter, particularly the ORF coverage. Furthermore, the combination of ORF-related features show a better performance than other two ORF-unrelated feature groups (Fig. 1B). In addition, ORF-related features are known to have great classification performance for the full-length transcripts (Fig. 1C,D), thus four existing ORF features and a new one (hexamer score distance) were selected in lncScore.

Then, we ranked features in the order of importance to the classification performance of lncScore on each testing datasets. The feature with the largest classification performance on each testing datasets was sorted at the first position, and then the feature with the biggest performance improvement (or the smallest performance decline) to the model with features sorted before it was ranked next. As shown in Supplementary Fig. S4, it is obvious that some features (e.g. ORF length, MCSS length, and MCSS coding score) significantly result in performance degradation on the partial-length testing datasets, while fewer features cause a slight performance decline on the full-length testing datasets. It can be seen that there is always an exon feature ranked in the first three features for each testing datasets. And for all the testing datasets, there are always two ORF features, one Exon feature, and one MCSS feature in the first four features, which proves that features derived from ORF, exon, and MCSS are complementary to each other. ORF length ranks first on the full-length testing datasets and ranks last on the partial-length testing datasets, which is due to the degradation of the ORF prediction accuracy for partial-length protein-coding transcripts. All of the exon features improved the performance on the mouse testing datasets, while exon GC-content improved the performance and the remaining two exon features degraded the performance on the human full-length testing datasets, which is contrary to that on the human partial-length testing dataset. All of these results suggest that exon features is a very useful feature for the identification of lncRNAs and is commentary to ORF-related features. Through sorting features, it can be seen that features make different contributions to lncScore on different kinds (partial- or full-length) of testing datasets. By using all of the features, lncScore shows a good performance on both the partial- and full-length testing datasets.

With the increasing number of annotated lncRNAs in GENCODE, the classifier would need to be updated, so the performance of building a new model is critical. Furthermore, with more and more novel lncRNAs discovered, new classifiers may need to be built for other species. In previous tools (e.g. CONC, CPC, CNCI, PLEK), support vector machine with a radial basis functional kernel (SVM-RBF) was widely used to build classifiers; however, using grid search to find the best parameter c and g for an optimal SVM-RBF model is very time consuming, particularly with a larger training dataset. In contrast, logistic regression (LR) model used is easier and faster to update and build, as shown in Supplementary Table S3, and it is generally more interpretable than SVM. We compared the modeling time for a LR model with that for a SVM-RBF model on the training datasets of human and mouse, and the result shows that the LR model cost less time than the SVM-RBF model. In addition, we also compared the classification performance (AUC) of logistic regression models and SVM-RBF models on the testing datasets, and the results showed that the AUC of logistic regression models are all larger than that of SVM-RBF models (Supplementary Table S4)^56^. Besides SVM-RBF, we also compared LR with an ensemble classifier – libD3C^57^. As shown in Supplementary Table S5, LR is slightly better than libD3C on the classification performance, but it is much faster than libD3C to build and test model. Therefore, logistic regression was selected to build lncScore model.

To optimize the execution performance for large-scale transcriptome data from RNA-seq, the multiprocessing module in Python’s standard library was used in lncScore to implement multithreading. CNCI and PLEK can also run in multi-threading manner, and in their programs, the same number of transcripts was assigned to each thread. However, CNCI and PLEK with 12 threads were only about 7 and 6 times (not 12 times) faster than with a single thread for the human full-length testing dataset, respectively, because tasks in each thread cannot be accomplished simultaneously and the total computing time depends on the finally completed thread. It was found that the running time of each thread mainly depends on the total length of the assigned transcripts. Thus, in our program, transcripts that were assigned to each thread have the same total transcripts length. Then, for the same datasets mentioned above, 12-threaded lncScore is nearly 11 times faster than single-threaded one and the running time decreases exponentially with the number of threads normally (Supplementary Fig. S5).

**Figure 4:**
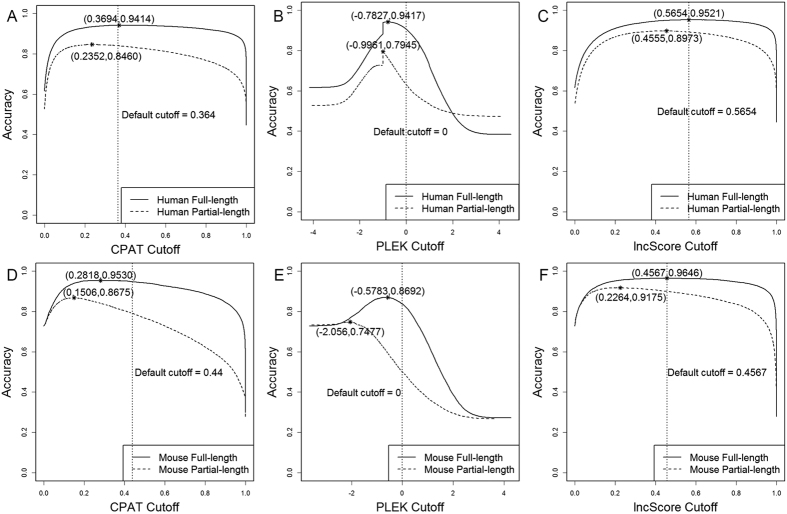
Accuracy versus cutoff score for testing datasets. The highest point of each line was marked with an asterisk.

To have a better trade-off between sensitivity and specificity, the cutoff score with the best accuracy against the full-length testing datasets was selected as the optimal one, with which lncScore outperformed other tools on all of the testing datasets. Moreover, when using the cutoff score with the best accuracy against the partial-length testing datasets, lncScore also showed a better performance than other tools (Supplementary Table S6). Compared with the default cutoff score, the new one derived from partial-length testing datasets leads to a higher sensitivity and NPV on all of the testing datasets, which means that lncScore can predict lncRNAs more precisely. Furthermore, we analyzed the cutoff score’s effect trend and extent to the overall accuracy of lncScore, and the same thing was also done for CPAT and PLEK. As shown in Fig. 4, the accuracy of lncScore changes slightly over a considerable range of cutoff score on all of the testing datasets, except for the mouse partial-length one, however, on which lncScore still performed better than other tools. CPAT presents a similar performance with lncScore on the human testing datasets, whereas it performs worse on mouse testing datasets. For PLEK, its accuracy changes dramatically with the cutoff score. These results suggested that lncScore is a stable and robust classifier, whose performance is relatively less affected by the cutoff score.

## Conclusion

In conclusion, with 11 exon-, MCSS-, and ORF-related features, we developed a novel alignment-free tool – lncScore – using a logistic regression model. Compared with existing alignment-free tools (e.g. CPAT, CNCI, and PLEK), lncScore showed much better performance on the human and mouse partial-length testing datasets. In addition, it also showed improved performance on the full-length testing datasets of human, mouse and five other species. lncScore can be run in a multithreaded manner, with much faster than competing approaches. Thus, lncScore is a fast, accurate, stable and robust tool for distinguishing protein-coding and long noncoding transcripts from RNA-seq data for many species.

## Additional Information

**How to cite this article**: Zhao, J. *et al*. lncScore: alignment-free identification of long noncoding RNA from assembled novel transcripts. *Sci. Rep.* **6**, 34838; doi: 10.1038/srep34838 (2016).

## Supplementary Material

Supplementary Information

## Figures and Tables

**Table 1:** Features used in lncScore.

	Feature Group		
	Exon	MCSS	ORF
Features (Acronym)	Hexamer Score (HS)	Length (L)	Length (L) & Coverage (C)
	Hexamer Score Distance (HSD)	Coding Score (CS)	Fickett Score (FS)
		Coding Score Percentage(CSP)	Hexamer Score (HS)
	GC-content (GC-c)		Hexamer Score Distance (HSD)

MCSS is the abbreviation of maximum coding subsequence.

**Table 2:** The area under ROC curve (%) of each single feature.

	Exon			MCSS			ORF				
	HS	HSD	GC-c	CS	L	CSP	L	C	FS	HS	HSD
HP	90.19	87.44	73.91	88.61	87.30	89.22	83.41	88.59	79.67	87.16	80.85
HF	90.92	87.59	81.67	96.45	96.19	95.16	97.06	85.44	81.87	90.48	84.84
MP	91.13	89.15	75.67	89.63	89.00	91.73	83.70	92.67	79.23	89.08	80.79
MF	92.47	89.85	83.50	96.94	97.06	96.51	97.51	89.66	81.40	92.99	85.13

The performance of each single feature from three different feature groups (e.g. ORF, exon, MCSS) was evaluated using AUC on the Partial Testing Datasets (HP & MP) and the Full Testing Datasets (HF & MF) of human and mouse species. The full name of the abbreviation of each feature was shown in the Table 1.

**Table 3:** Performance (%) comparison on the partial- and full-length testing dataset.

		Partial-length testing dataset				Full-length testing dataset			
		CPAT	CNCI	PLEK	lncScore	CPAT	CNCI	PLEK	lncScore
Human	Accuracy	84.03	80.51	63.14	89.12	94.41	92.20	90.61	95.21
	Sensitivity	76.19	65.40	31.76	84.15	94.97	89.00	85.96	95.56
	PPV	92.12	96.46	94.83	94.61	95.46	98.16	98.62	96.64
	Specificity	92.75	97.33	98.07	94.67	92.75	97.33	98.07	94.67
	NPV	77.78	71.65	56.36	84.29	92.00	84.64	81.31	92.99
	MCC	69.41	65.36	39.07	78.85	87.59	84.55	81.96	89.93
Mouse	Accuracy	79.04	76.47	50.07	89.92	94.65	92.83	83.67	96.46
	Sensitivity	72.88	69.24	35.34	88.39	94.19	91.56	81.17	97.35
	PPV	97.97	98.05	90.91	97.61	98.39	98.48	95.75	97.78
	Specificity	95.88	96.23	90.35	94.08	95.88	96.23	90.35	94.08
	NPV	56.40	63.37	33.82	74.78	86.05	80.98	64.18	92.99
	MCC	61.15	58.02	25.21	77.27	84.21	83.52	65.47	91.10

The default cutoff of CPAT, PLEK, and lncScore is shown in Fig. 4, and the default cutoff of CNCI is 0.

**Table 4:** The overall ACC and AUC (%) of CPAT, CNCI, PLEK, lncScore, and 10-fold cross validation on 5 other species datasets.

	Zebrafish		Fruitfly		C. elegans		Rat		Sheep	
	ACC	AUC	ACC	AUC	ACC	AUC	ACC	AUC	ACC	AUC
CPAT*	78.51	82.54	95.52	98.36	*	*	*	*	*	*
CPAT^H^	78.66	83.17	92.80	98.36	97.55	99.66	89.25	94.22	77.58	88.03
CPAT^M^	78.29	82.54	94.44	98.37	96.53	99.69	89.72	94.23	79.99	87.70
CNCI	69.49	77.66	86.08	95.00	64.33	83.89	81.32	88.70	84.77	85.33
PLEK	62.32	70.46	82.19	89.90	75.98	95.27	83.23	89.80	66.55	69.90
lncScore^H^	79.25	84.95	95.54	98.67	96.41	99.33	89.28	94.41	84.77	94.28
lncScore^M^	79.84	85.43	96.44	98.88	97.28	99.35	89.27	94.54	82.20	93.73
10_CV	79.91	86.97	96.66	98.64	98.23	99.21	89.41	93.80	92.78	96.96

CPAT^*^ represents CPAT models for zebrafish and fly. CPAT^H^ and CPAT^M^ stand for CPAT models for human and mouse, respectively. lncScore^H^ and lncScore^M^ refer to the models of lncScore respectively for human and mouse. 10_CV is the abbreviation of 10-fold cross validation.
