# Supplementary material for: lncScore: alignment-free identification of long noncoding RNA from assembled novel transcripts
Source: Sci Rep. 2016 Oct 6;6:34838. doi: 10.1038/srep34838 (PMC5052565; doi:10.1038/srep34838)
Supplement: Supplementary Information [file srep34838-s1.pdf]

lncScore: alignment-free identification of long noncoding RNA from assembled novel transcripts

Jian Zhao[1,2], Xiaofeng Song [1,*], Kai Wang[2, 3,*]

[1]Department of Biomedical Engineering, Nanjing University of Aeronautics and Astronautics, Nanjing 210016, China

[2]Zilkha Neurogenetic Institute, Keck School of Medicine, University of Southern California, Los Angeles, CA 90089, USA

[3]Division of Bioinformatics, Department of Preventive Medicine, Keck School of Medicine, University of Southern California, Los Angeles, CA 90089, USA

*Corresponding authors: xfsong@nuaa.edu.cn; kaiwang@usc.edu

Table S1. List of alignment-free methods for the classification of coding/noncoding transcripts

Method name	Algorithm	Number of features	Programming language	Multithreading
CPAT	Logistic regression	4	Python	F
CNCI	SVM	5	Python	T
PLEK	SVM	1,364	Python	T
lncRNA-MFDL	Deep learning	138	Python + MATLAB	F
lncScore	Logistic regression	11	Python	T

T represents ‘True’, and F represents ‘False’.

Table S2. Numbers of the protein-coding and long noncoding transcripts in the testing dataset of other species

	Protein-coding transcripts	Long noncoding transcripts
Zebrafish	2711	2711
Fruitfly	2723	2723
C. elegans	1615	1615
Rat	3163	3163
Sheep	2009	2009

For long noncoding transcripts, only those transcripts labeled with “lncRNA”, “ncRNA”, “antisense”, “sense_intronic”, “sense_overlapping”, or “processed_transcript” were selected, and the same number of protein coding transcripts were randomly selected from the transcripts labeled with “ensemble:known”, “flybase:known”, or “wormbase:known”. All of the transcripts were derived from the Ensembl database (release 82).

Table S3. Comparison of the running time of CPAT, PLEK and lncScore for building a classification model

	CPAT	PLEK	lncScore		
	LR	SVM-RBF	LR	LR^{12}	SVM-RBF
HT	0.228	309.667	1.448	0.217	53.150
MT	0.880	2657.485	7.926	0.901	481.838

Running time (minutes) was test on the human (HT) and mouse (MT) training datasets.

LR represents logistic regression, and LR^{12} indicates that LR model was built with 12 threads running.

Table S4. AUC (%) comparison of LR and SVM-RBF model

		HP	HF	MP	MF
lncScore	LR	95.47	98.60	96.63	99.05
	SVM-RBF	94.73	98.41	95.89	99.02

The performance of LR and SVM-RBF model was evaluated using AUC on the Partial Testing Datasets (HP & MP) and the Full Testing Datasets (HF & MF) of human and mouse species.

The best c and g for human/mouse SVM-RBF model are 8192/32768 and 0.03125/0.03125.

Table S5. Comparison of LR, libSVM and libD3C on AUC, training & testing time (seconds)

		HP	HF	MP	MF
AUC	LR	0.955	0.986	0.966	0.990
	libSVM	0.762	0.798	0.807	0.847
	libD3C	0.947	0.985	0.961	0.992
Training time	LR	0.12	0.14	0.59	0.7
	libSVM	17.01	18.3	310.91	315.74
	libD3C	24.29	23.18	286.41	290.21
Testing time	LR	0.21	0.25	0.09	0.14
	libSVM	58.07	73.7	69.69	69.17
	libD3C	127.11	161.71	74.83	73.56

The performance was evaluated on the Partial Testing Datasets (HP & MP) and the Full Testing Datasets (HF & MF) of human and mouse species. The LR, libSVM, and libD3C models were trained and tested by using the latest Weka 3, which is a data mining software in java. The time taken to calculated features of transcripts was not included in the training and testing time.

Table S6. Performance (%) comparison on the testing dataset

			CPAT	CNCI	PLEK	lncScore[1]	lncScore[2]
Human	Partial-length	Cutoff	0.364	0	0	0.4555	0.5654
		Accuracy	84.03	80.51	63.14	89.73	89.12
		Sensitivity	76.19	65.40	31.76	87.47	84.15
		PPV	92.12	96.46	94.83	92.62	94.61
		Specificity	92.75	97.33	98.07	92.24	94.67
		NPV	77.78	71.65	56.36	86.86	84.29
		MCC	69.41	65.36	39.07	79.60	78.85
	Full-length	Accuracy	94.41	92.20	90.61	94.89	95.21
		Sensitivity	94.97	89.00	85.96	96.54	95.56
		PPV	95.46	98.16	98.62	95.23	96.64
		Specificity	92.75	97.33	98.07	92.24	94.67
		NPV	92.00	84.64	81.31	94.33	92.99
		MCC	87.59	84.55	81.96	89.18	89.93
			CPAT	CNCI	PLEK	lncScore[1]	lncScore[2]
Mouse	Partial-length	Cutoff	0.44	0	0	0.2264	0.4567
		Accuracy	79.04	76.47	50.07	91.75	89.92
		Sensitivity	72.88	69.24	35.34	93.56	88.39
		PPV	97.97	98.05	90.91	95.08	97.61
		Specificity	95.88	96.23	90.35	86.77	94.08
		NPV	56.40	63.37	33.82	83.15	74.78
		MCC	61.15	58.02	25.21	79.27	77.27
	Full-length	Accuracy	94.65	92.83	83.67	95.44	96.46
		Sensitivity	94.19	91.56	81.17	98.68	97.35
		PPV	98.39	98.48	95.75	95.23	97.78
		Specificity	95.88	96.23	90.35	86.77	94.08
		NPV	86.05	80.98	64.18	96.09	92.99
		MCC	87.21	83.52	65.47	88.33	91.10

lncScore[1] represents lncScore using the cutoff score with the best accuracy against the partial-length testing datasets, while lncScore[2] represents lncScore using the cutoff score with the best accuracy against the full-length testing datasets.

Figure S1. Processes of building the human training and testing datasets used in lncScore.

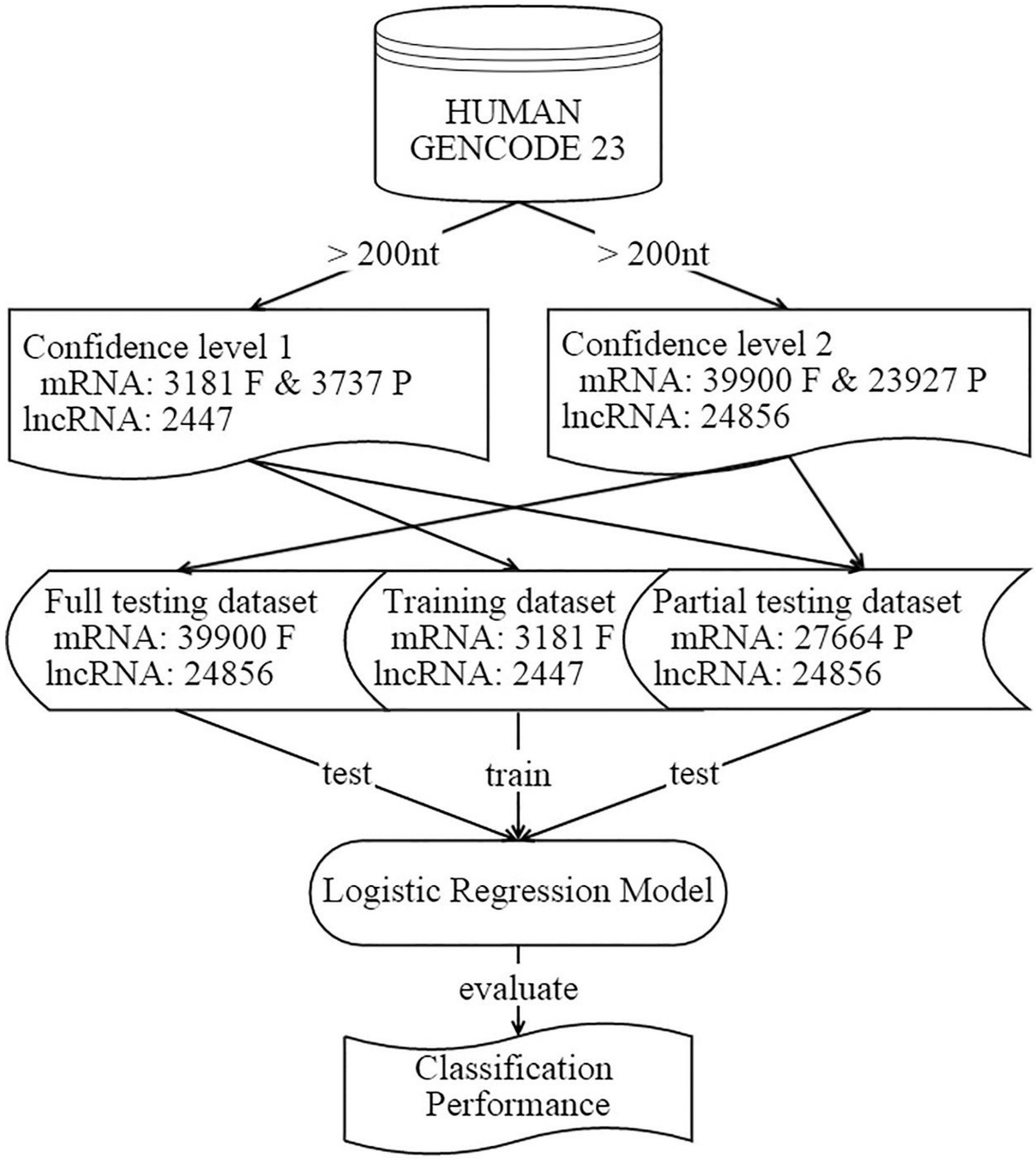

F represents full-length transcripts, and *P* represents partial-length transcripts.

Figure S2. Processes of building the mouse training and testing datasets used in lncScore.

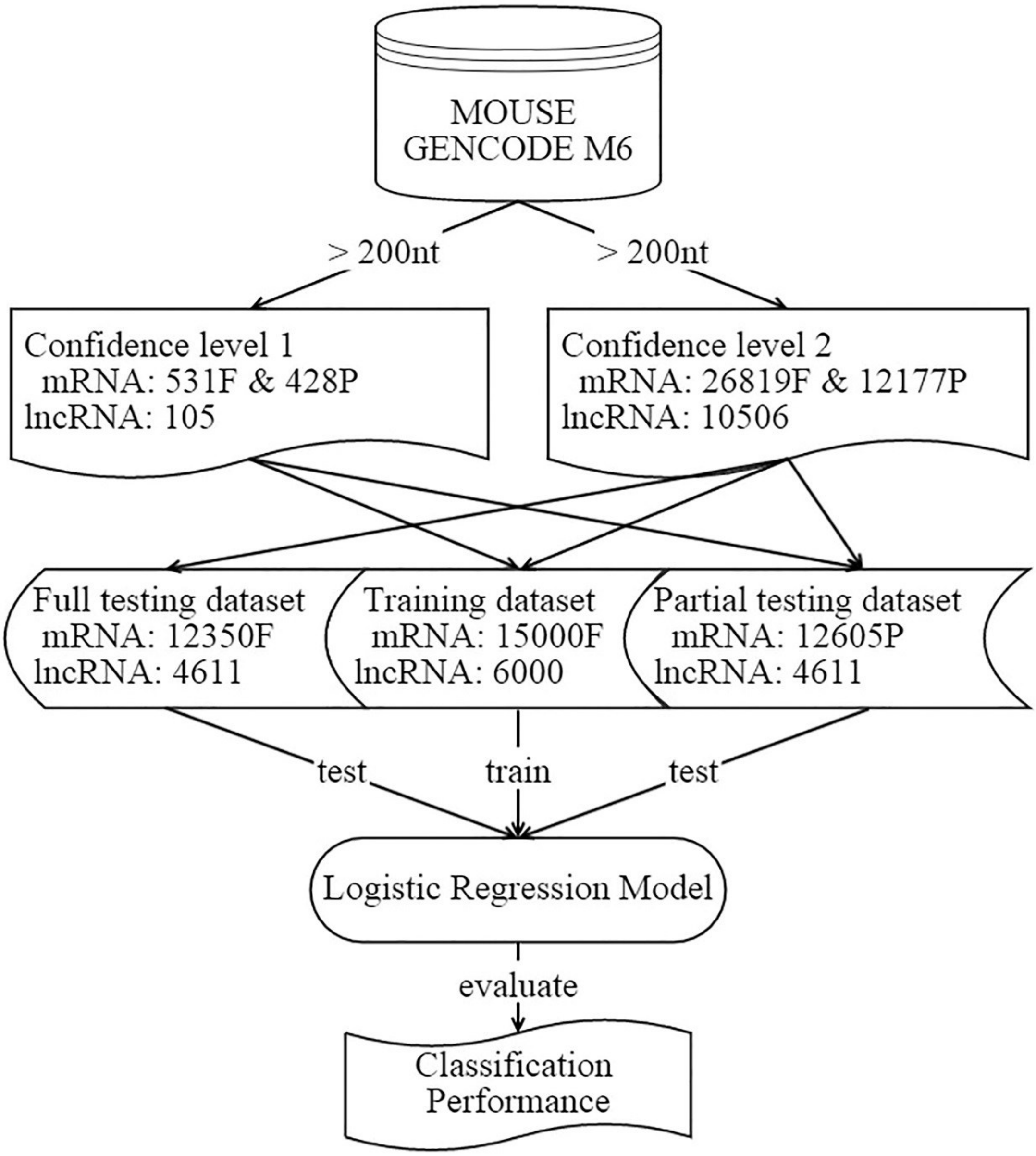

F represents full-length transcripts, and P represents partial-length transcripts.

Figure S3. ROC curves of 10-fold cross validation using different feature groups on the training datasets

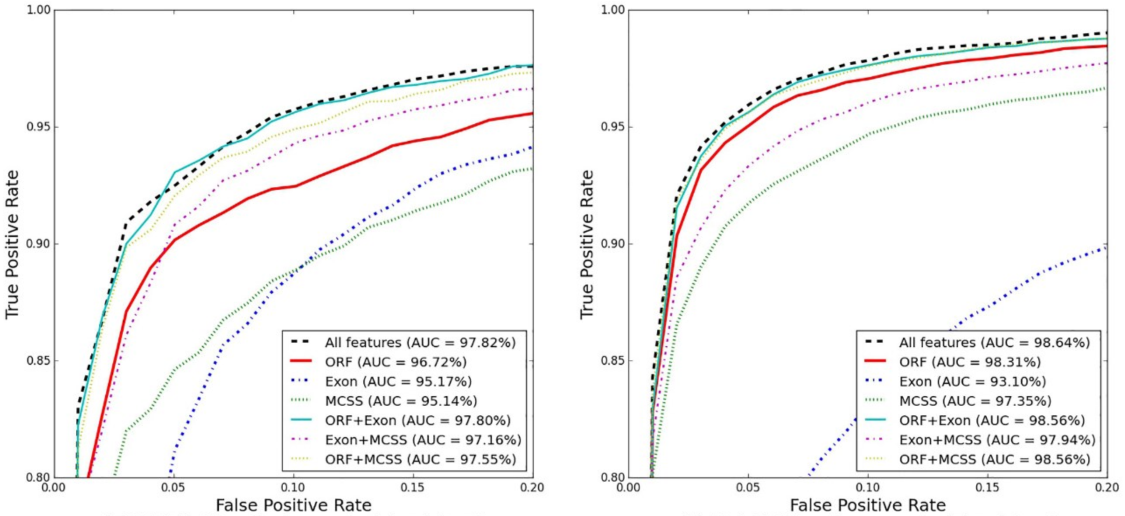

(a) 10-fold CV on the human training dataset

(b) 10-fold CV on the mouse training dataset

Figure S4. The contribution of each feature to the final performance of lncScore on each testing datasets

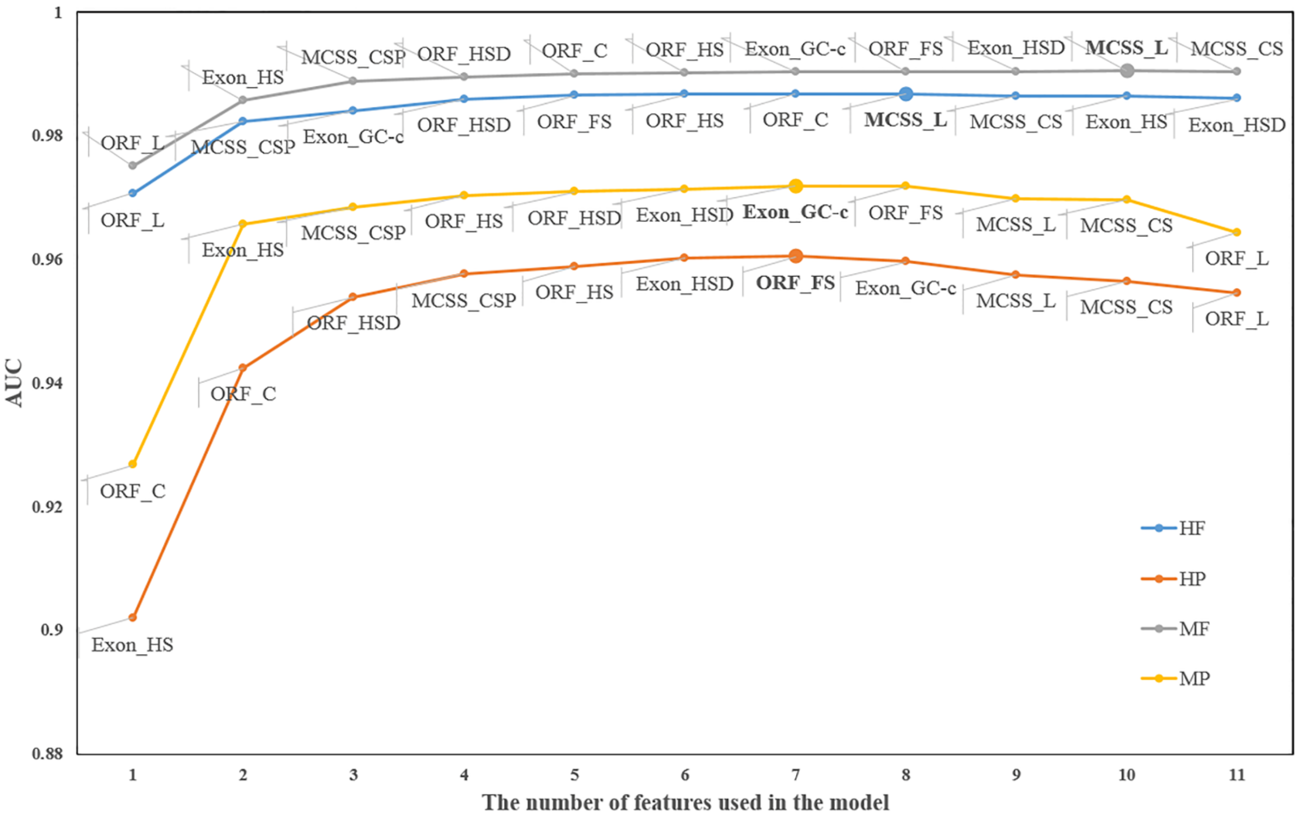

The feature with the biggest performance increases (or the smallest performance decreases) was added to the logistic model each time. Then performance was evaluated using AUC on the Partial Testing Datasets (HP & MP) and the Full Testing Datasets (HF & MF) of human and mouse species, individually. The full name of the abbreviation of each feature was shown in the Table 1.

The maximum AUC on each testing datasets was labeled with a larger dot.

Figure S5. Running time of lncScore with different threads

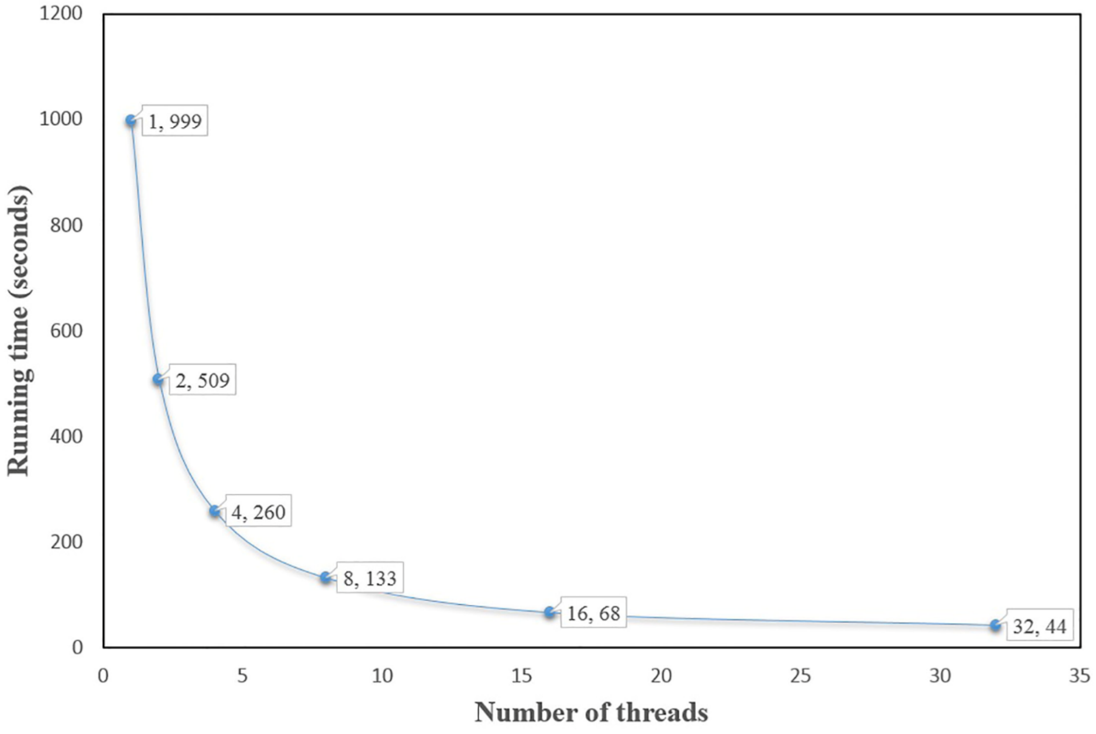

The total computing time of lncScore was measured on the human full-length testing dataset with four 3.30GHz Intel Xeon E5-4627 processors, 1 TB memory and Linux operating system.
